# Supplementary material for: ADAR1 haploinsufficiency and sustained picornaviral RdRp dsRNA synthesis synergize to dysregulate RNA editing and cause multi-system interferonopathy
Source: bioRxiv. 2025 May 28:2025.01.21.634124. Originally published 2025 Jan 22. Preprint. [Version 2] doi: 10.1101/2025.01.21.634124 (PMC11785089; doi:10.1101/2025.01.21.634124)
Supplement: Supplement 1 [file NIHPP2025.01.21.634124v2-supplement-1.pdf]

bioRxiv preprint doi: https://doi.org/10.1101/2025.01.21.634124; this version posted May 28, 2025. The copyright holder for this preprint (which was not certified by peer review) is the author/funder, who has granted bioRxiv a license to display the preprint in perpetuity. It is made available under aCC-BY 4.0 International license.

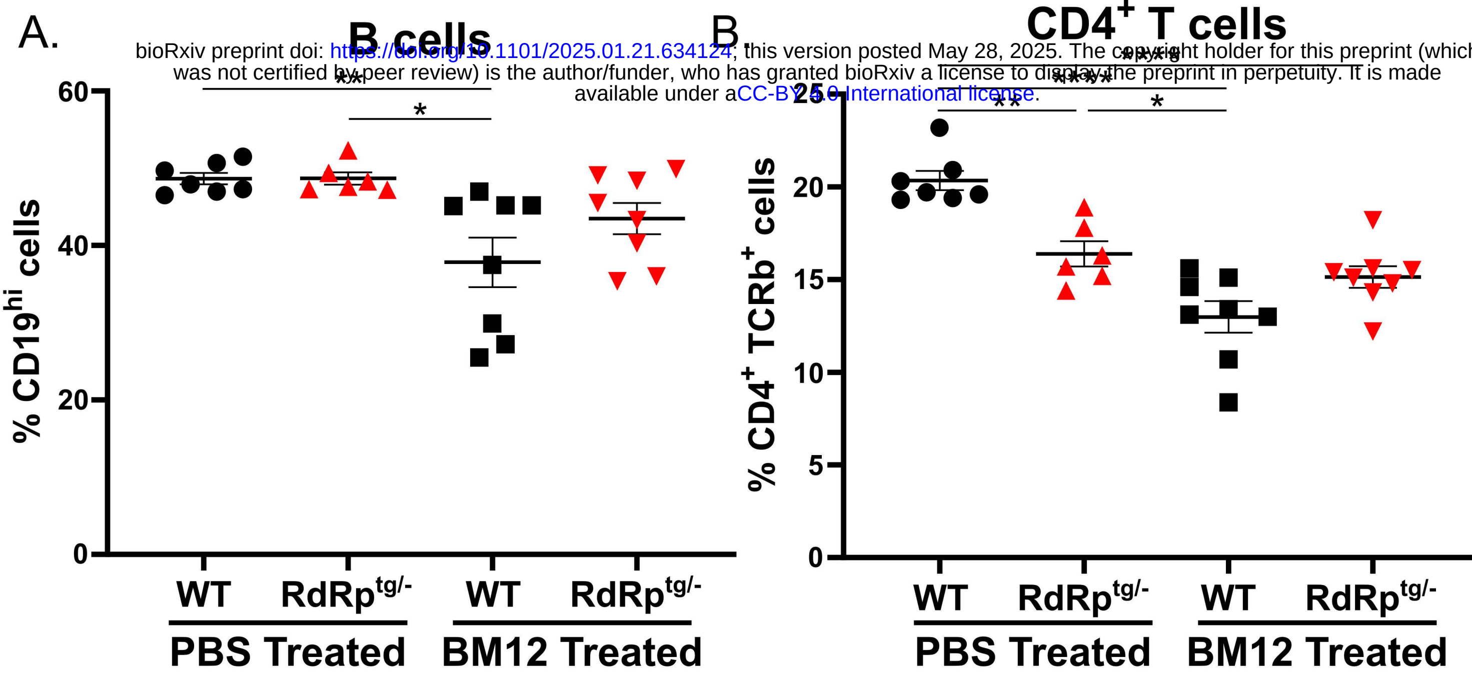

Supplemental Figure 1

A

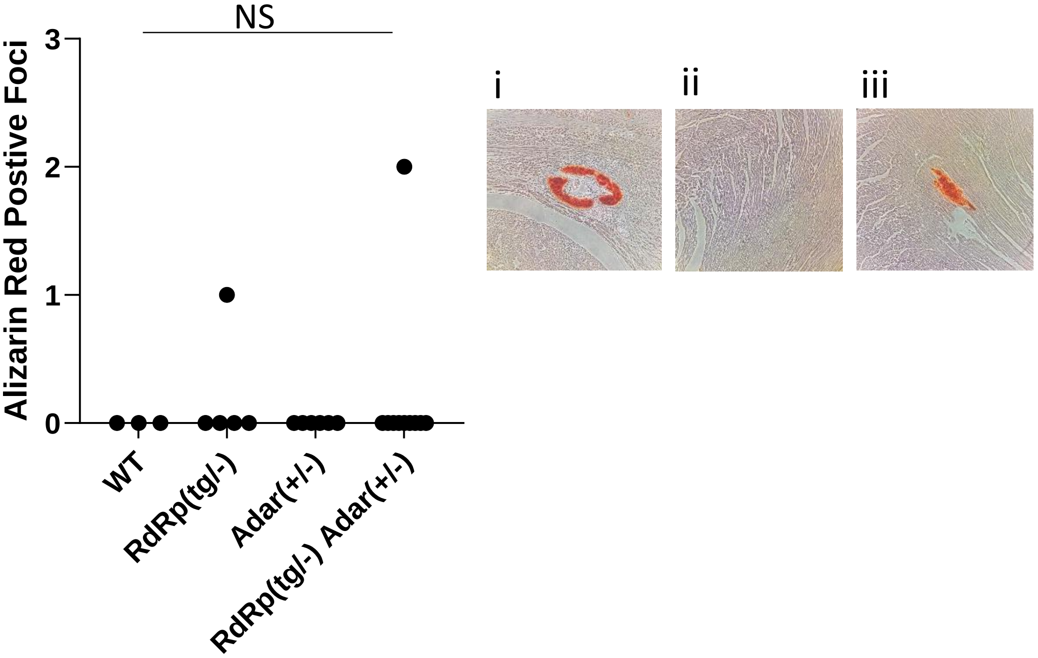

B

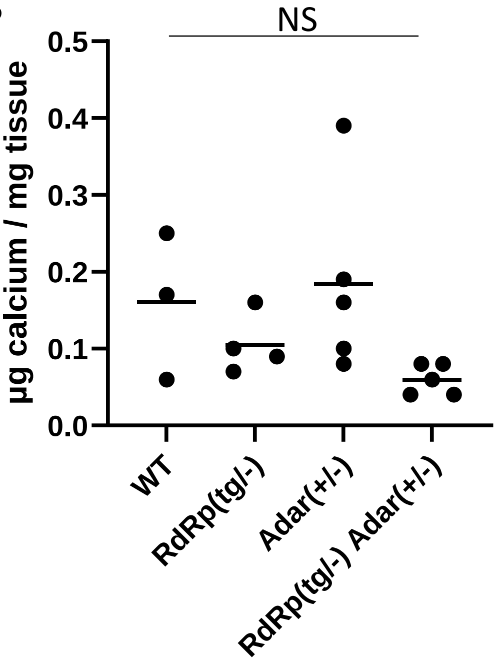

Supplemental Figure 2

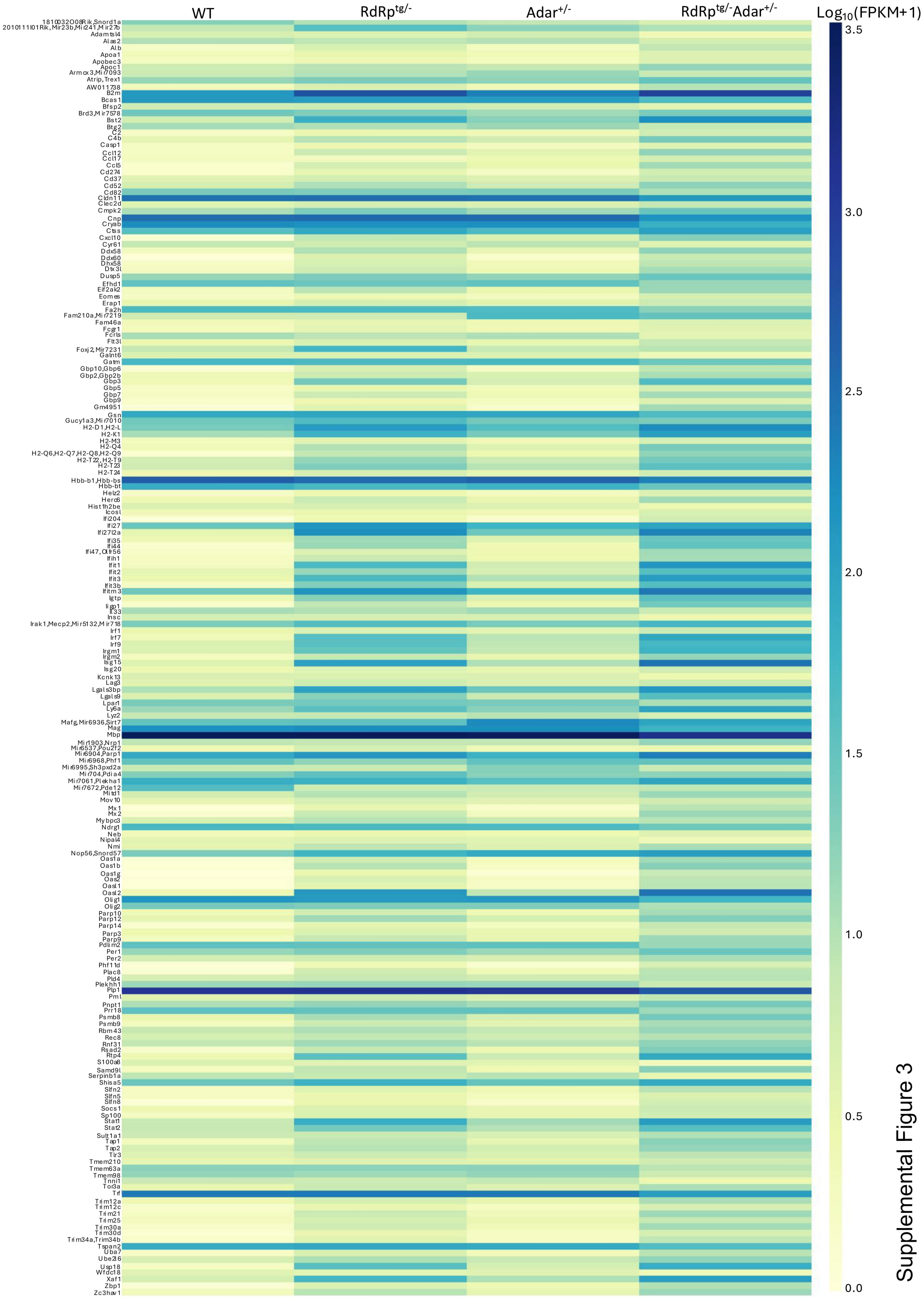

Supplemental Figure 3

bioRxiv preprint doi: https://doi.org/10.1101/2023.01.[illegible] [illegible] posted [illegible] 2023. The copyright holder for this preprint (which was not certified by peer review) is the author/funder, who has granted bioRxiv a license to display the preprint in perpetuity. It is made available under aCC-BY 4.0 International license.

	WT vs. RdRP		WT vs. ADAR		WT vs. ADAR-RdRP		RdRP vs. ADAR		RdRP vs. ADAR-RdRP		ADAR vs. ADAR-RdRP	
**Diseases or Functions Annotation**	**-log(B-H p-value)**	**z-score**	**-log(B-H p-value)**	**z-score**	**-log(B-H p-value)**	**z-score**	**-log(B-H p-value)**	**z-score**	**-log(B-H p-value)**	**z-score**	**-log(B-H p-value)**	**z-score**
Antimicrobial response	0.00000	**2.39**	1.97E-09	1.455	NaN	NaN	0.00000	**-2.39**	1.08E-05	NaN	1.21E-12	0.995
Antiviral response	0.00000	**2.39**	2.77E-11	1.455	NaN	NaN	0.00000	**-2.39**	2.82E-06	NaN	6.75E-12	0.995
Apoptosis of breast cancer cell lines	NaN	NaN	NaN	NaN	NaN	NaN	NaN	NaN	NaN	NaN	0.00146	**2.808**
Apoptosis of epithelial cells	NaN	NaN	NaN	NaN	NaN	NaN	NaN	NaN	NaN	NaN	0.00003	**2.55**
Apoptosis of liver cells	NaN	NaN	NaN	NaN	NaN	NaN	NaN	NaN	NaN	NaN	0.00146	**2.169**
Cell death of epithelial cells	0.00251	**2.414**	NaN	NaN	NaN	NaN	NaN	NaN	1.06E-03	0.922	1.41E-05	**3.058**
Demyelination of nerves	NaN	NaN	NaN	NaN	NaN	NaN	NaN	NaN	0.00001	**2.195**	NaN	NaN
Differentiation of Th17 cells	NaN	NaN	NaN	NaN	NaN	NaN			NaN	NaN	0.00014	**-2.382**
Encephalitis	3.09E-04	-1.925	3.72E-07	0.264	NaN	NaN	NaN	NaN	1.09E-04	-1.361	0.00000	**-3.488**
Experimental autoimmune encephalomyelitis	5.09E-04	-1.639	7.57E-07	0.659	NaN	NaN	1.45E-06	1.881	1.66E-04	-1.37	0.00000	**-3.326**
Inflammation of absolute anatomical region	8.46E-06	-1.905	5.51E-08	-0.527	NaN	NaN	1.39E-07	1.365	0.00002	**-2.537**	0.00000	**-3.351**
Inflammation of organ	3.08E-05	-1.264	1.75E-07	0.255	NaN	NaN	1.71E-07	1.078	0.00001	**-2.346**	0.00000	**-2.881**
Inhibition of cells	NaN	NaN	NaN	NaN	NaN	NaN	NaN	NaN	NaN	NaN	0.00142	**2.166**
Maturation of phagocytes	NaN	NaN	2.29E-03	-0.169	NaN	NaN	0.00074	**-2.183**	NaN	NaN	5.47E-04	0.891
Necrosis	8.48E-03	1.117	NaN	NaN	NaN	NaN	NaN	NaN	0.00160	**2.156**	0.00004	**2.291**
Necrosis of epithelial tissue	NaN	NaN	NaN	NaN	NaN	NaN	NaN	NaN	1.36E-04	1.014	0.00000	**2.072**
Neuronal cell death	NaN	NaN	NaN	NaN	NaN	NaN	NaN	NaN	0.00476	**2.302**	0.00006	**2.309**
Proliferation of antigen presenting cells	NaN	NaN	NaN	NaN	NaN	NaN	2.26E-03	-0.865	3.64E-03	NaN	0.00014	**2.201**
Proliferation of blood cells	0.00421	**2.084**	NaN	NaN	NaN	NaN	NaN	NaN	9.98E-04	0.317	8.85E-06	0.535
Proliferation of immune cells	0.00429	**2.084**	NaN	NaN	NaN	NaN	NaN	NaN	9.14E-04	0.358	1.70E-05	0.808
Quantity of blood cells	0.00064	**2.383**	NaN	NaN	NaN	NaN	0.00193	**-2.021**	1.63E-04	0.487	2.79E-06	0.67
Quantity of IL-12 in blood	0.00002	**-2**	6.62E-03	NaN	NaN	NaN	0.00002	**2**	NaN	NaN	0.00007	**-2**
Quantity of interleukin	NaN	NaN	NaN	NaN	NaN	NaN	NaN	NaN	NaN	NaN	0.00064	**-2**
Quantity of leukocytes	0.00046	**2.295**	NaN	NaN	NaN	NaN	1.60E-03	-1.906	1.02E-04	0.167	9.96E-07	0.336
Quantity of phagocytes	0.00104	**2.191**	NaN	NaN	NaN	NaN	2.21E-03	-1.544	2.25E-05	-0.125	5.80E-05	-0.102
Quantity of protein in blood	0.00769	**-2.909**	NaN	NaN	NaN	NaN	0.00034	**2.088**	NaN	NaN	6.48E-04	-1.717
Rheumatic Disease	0.00000	**-2.219**	7.94E-05	-1	NaN	NaN	7.74E-07	1.179	3.07E-05	-0.503	2.99E-08	-1.157

Supplemental Figure 4

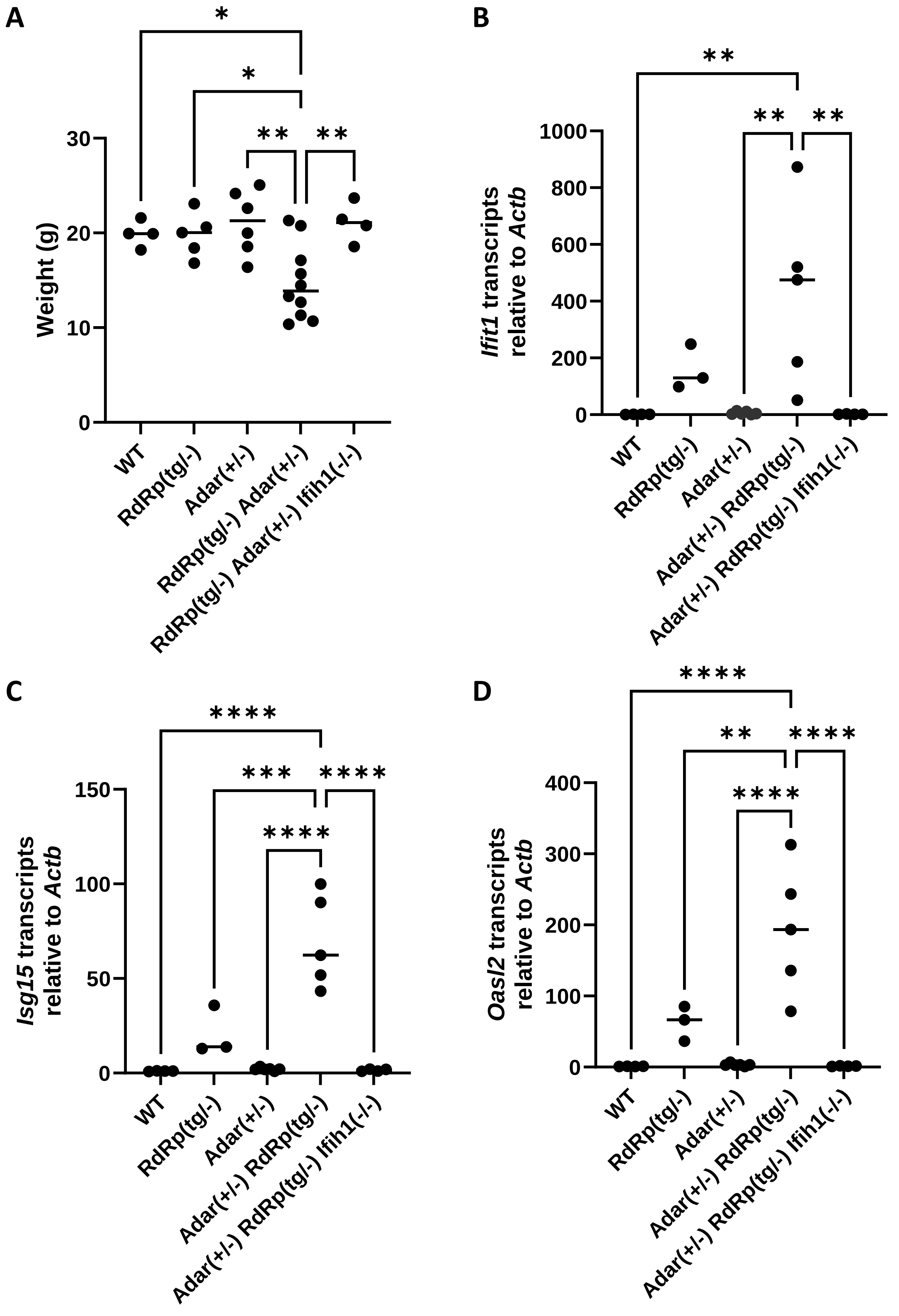

Supplemental Figure 5

bioRxiv preprint doi: https://doi.org/10.1101/2025.01.21.634124; this version posted May 28, 2025 ... was not certified by peer review) is the author/funder, who has granted bioRxiv a license to dis... available under aCC-BY 4.0 International license.

Diseases or Functions Annotation	WT vs. RdRp$^{tg/-}$	WT vs. Adar$^{+/-}$	WT vs. RdRp$^{tg/-}$ Adar$^{+/-}$	RdRp$^{tg/-}$ vs. Adar$^{+/-}$	RdRp$^{tg/-}$ vs. RdRp$^{tg/-}$ Adar$^{+/-}$	Adar$^{+/-}$ vs. RdRp$^{tg/-}$ Adar$^{+/-}$
Activation of cells	[illegible]	[illegible]	[illegible]	[illegible]	[illegible]	[illegible]
Activation of macrophages	1.803	1.282	2.145	-1.803	1.898	1.633
Activation of myeloid cells	1.472	0.943	2.084	-1.472	2.095	1.698
Activation of phagocytes	1.469	0.938	1.765	-1.469	[illegible]	1.795
Adhesion of blood cells	1.989	NA	2.637	NA	2.132	2.025
Antigen presentation	2.414	NA	2.414	NA	NA	2.414
Antigen presentation by cells	2	NA	2	NA	NA	2
Antimicrobial response	2.787	2.406	2.792	-2.947	NA	2.792
Antiviral response	2.787	2.406	2.792	-2.947	NA	2.792
Antiviral response of cells	2.219	1.982	1.31	-2.219	NA	1.31
Apoptosis	0.916	0.197	1.616	-0.696	1.157	2.443
Apoptosis of tumor cell lines	NA	NA	1.173	-0.889	NA	2.09
Autophagy of cells	2.072	NA	1.363	-1.502	1.095	1.152
Cell cycle progression	2.961	NA	2.195	-2.809	NA	2.448
Cell death of epithelial cells	NA	NA	1.966	NA	NA	2.68
Cell death of tumor cell lines	1.044	NA	1.396	-1.388	NA	2.158
Cell movement of leukocytes	2.904	NA	1.35	-2.394	1.501	1.387
Cell movement of monocytes	2.185	NA	2.083	NA	1.571	2.083
Cell movement of T lymphocytes	2.544	1.948	1.072	-1.824	1.645	1.072
Cellular homeostasis	1.314	1.026	2.089	-1.662	0.957	1.356
Cellular infiltration	2.619	NA	0.842	-1.692	1.402	1.042
Cellular infiltration by leukocytes	2.696	2.342	1.081	-2.13	1.481	1.294
Cellular infiltration by lymphocytes	2.187	NA	0.201	-1.507	0.692	0.201
Cellular infiltration by myeloid cells	1.878	2.091	0.83	NA	0.935	0.791
Contact growth inhibition	2.232	NA	2.467	-2.052	NA	2.467
Contact growth inhibition of tumor	NA	NA	2.022	-1.49	NA	2.022
Coordination	NA	NA	NA	NA	-2.401	NA
Cytostasis	2.442	NA	2.742	-2.522	NA	2.742
Cytostasis of tumor cell lines	1.972	1.451	NA	-1.762	NA	2.232
Cytotoxicity of cytotoxic T cells	2.439	NA	2.635	-1.988	NA	2.438
Cytotoxicity of lymphocytes	2.794	NA	2.605	-2.048	NA	2.412
Cytotoxicity of T lymphocytes	NA	NA	2.332	-1.583	NA	2.112
Degradation of DNA	NA	NA	2.781	NA	NA	2.781
Demyelination of nerves	NA	NA	1.798	NA	2.057	1.541
Differentiation of T lymphocytes	1.859	NA	1.227	-2.1	NA	0.815
Elimination of cells	NA	NA	2.2	NA	1.964	2.2
Encephalitis	-1.515	-1	-2.141	NA	-1.405	-2.141
Endocytosis	2.6	NA	NA	NA	NA	0.566
Fragmentation of DNA	NA	NA	2.598	NA	NA	2.598
Hematologic cancer	NA	NA	-2.752	NA	NA	-2.752
Hematologic cancer of cells	NA	NA	-2.396	NA	NA	-2.396
Hematopoietic neoplasm	NA	NA	-2.032	NA	NA	-2.032
Immune response of brain	-1.745	NA	-2.311	1.992	NA	-2.311
Immune response of cells	3.924	3.1	2.981	-3.066	NA	2.445
Immune response of leukocytes	2.415	1.981	0.755	-1.218	1.004	0.08
Infection by Flaviviridae	-1.732	-1.732	-2	1.732	NA	-2
Infection by Herpesviridae	NA	NA	-2.187	NA	NA	-2.187
Infection by RNA virus	-1.879	-2.019	-2.719	2.088	-1.388	-2.745
Infection of cells	-1.152	NA	-2.341	1.417	-1.2	-2.371
Infection of kidney cell lines	-1.651	-1.337	-2.093	1.651	NA	-2.291
Infection of mammalia	-3.48	-2.662	-3.452	3.388	-1.445	-3.207
Infiltration by T lymphocytes	2.187	NA	0.052	-1.507	0.842	0.052
Inflammation of absolute anatomic	-1.386	-0.851	-2.132	1.358	-0.958	-2.132
Inflammation of organ	-1.311	-0.729	-2.222	1.29	-0.76	-2.173
Inhibition of lymphocytes	NA	NA	2.172	NA	NA	2.172
Inhibition of RNA virus	1.974	NA	2.196	-1.974	NA	1.568
Inhibition of virus	NA	NA	2.415	-2.211	NA	1.848
Innate immune response	2.63	2.219	3.43	-3.132	2.8	3.43
Leukocyte migration	2.991	NA	1.565	-2.488	1.505	1.326
Lymphatic system tumor	NA	NA	-2.592	NA	NA	-2.592
Lymphocyte migration	2.721	2.144	0.753	-2.044	1.211	0.753
Lymphocytic cancer	NA	NA	-2.593	NA	NA	-2.593
Lymphocytic neoplasm	NA	NA	-2.593	NA	NA	-2.593
Lymphohematopoietic cancer	NA	NA	-2.752	NA	NA	-2.752
Lymphohematopoietic neoplasia	NA	NA	-2.032	NA	NA	-2.032
Lymphoid cancer	NA	NA	-2.593	NA	NA	-2.593
Lymphoreticular neoplasm	NA	NA	-2.4	NA	NA	-2.4
Migration of cancer cells	1.342	2	0.213	-1.342	0.956	0.508
Migration of cells	3.179	2.898	1.999	-2.573	0.825	1.285
Morbidity or mortality	-2.789	NA	-1.199	NA	0.575	-0.651
Necrosis	1.494	1.476	2.39	-1.714	2.274	2.989
Necrosis of epithelial tissue	NA	NA	1.737	NA	NA	2.346
Neoplasia of blood cells	NA	NA	-2.512	NA	NA	-2.512
Neoplasia of leukocytes	NA	NA	-2.396	NA	NA	-2.396
Neuromuscular disease	2.219	1.982	1.664	-1.982	1.387	1.664
Polarization of blood cells	NA	NA	NA	NA	2	NA
Polarization of cells	NA	1.982	NA	NA	2.219	NA
Production of protein	1.53	1.217	1.148	-2.096	0.485	0.678
Quantity of blood cells	2.217	1.36	0.079	-2.117	0.041	0.653
Quantity of CD8+ T lymphocyte	2.388	2.191	1.721	-2.207	1.461	1.721
Quantity of cytokine	NA	NA	-2.343	NA	NA	-2.154
Quantity of IFNG in blood	-2.183	NA	-2.4	2.183	NA	-2.4
Quantity of MHC Class I on cell surf	2.207	NA	2.207	-1.981	NA	2.207
Recruitment of leukocytes	2.339	2.339	2.156	-1.666	1.812	1.089
Recruitment of T lymphocytes	1.96	1.96	2.199	-1.96	1.996	NA
Replication of Flaviviridae	-2.643	-2.643	-2.988	2.826	-2.275	-2.988
Replication of flavivirus	-1.963	-2.181	-2.181	2.181	-1.939	-2.181
Replication of Hepatitis C virus	-2.335	-2.111	-2.528	2.324	NA	-2.528
Replication of Herpesviridae	-2.132	-2.828	-3.175	2.525	-2.236	-3.175
Replication of Murine herpesvirus 4	-2.236	-2	-2.828	2.236	NA	-2.828
Replication of RNA virus	-2.875	-3.077	-3.617	3.292	-2.304	-3.497
Replication of vesicular stomatitis v	-2.679	-2.473	-3.129	2.841	-1.702	-3.129
Replication of viral replicon	-2.818	-3.115	-3.56	3.271	-2.782	-3.56
Replication of virus	-3.082	-3.443	-3.924	3.583	-2.607	-3.812
Response of antigen presenting cel	2.219	NA	1.65	NA	NA	0.799
Response of embryonic cell lines	1.96	NA	2.4	-2.2	NA	2.4
Response of epithelial cell lines	1.969	NA	2.404	-2.207	NA	2.404
Response of kidney cell lines	1.969	NA	2.404	-2.207	NA	2.404
Response of phagocytes	2.207	2.207	NA	-2.207	NA	0.845
Transactivation of RNA	NA	1.961	NA	-2.41	NA	NA
Viral Infection	-3.615	-3.851	-4.1	3.801	-2.583	-3.943
Viral life cycle	NA	NA	-3.683	NA	NA	-3.683

Supplemental Figure 6

bioRxiv preprint doi: https://doi.org/10.1101/2025.01.21.634124; this version posted May 28, 2025. The copyright holder for this preprint (which was not certified by peer review) is the author/funder, who has granted bioRxiv a license to display the preprint in perpetuity. It is made available under aCC-BY 4.0 International license.

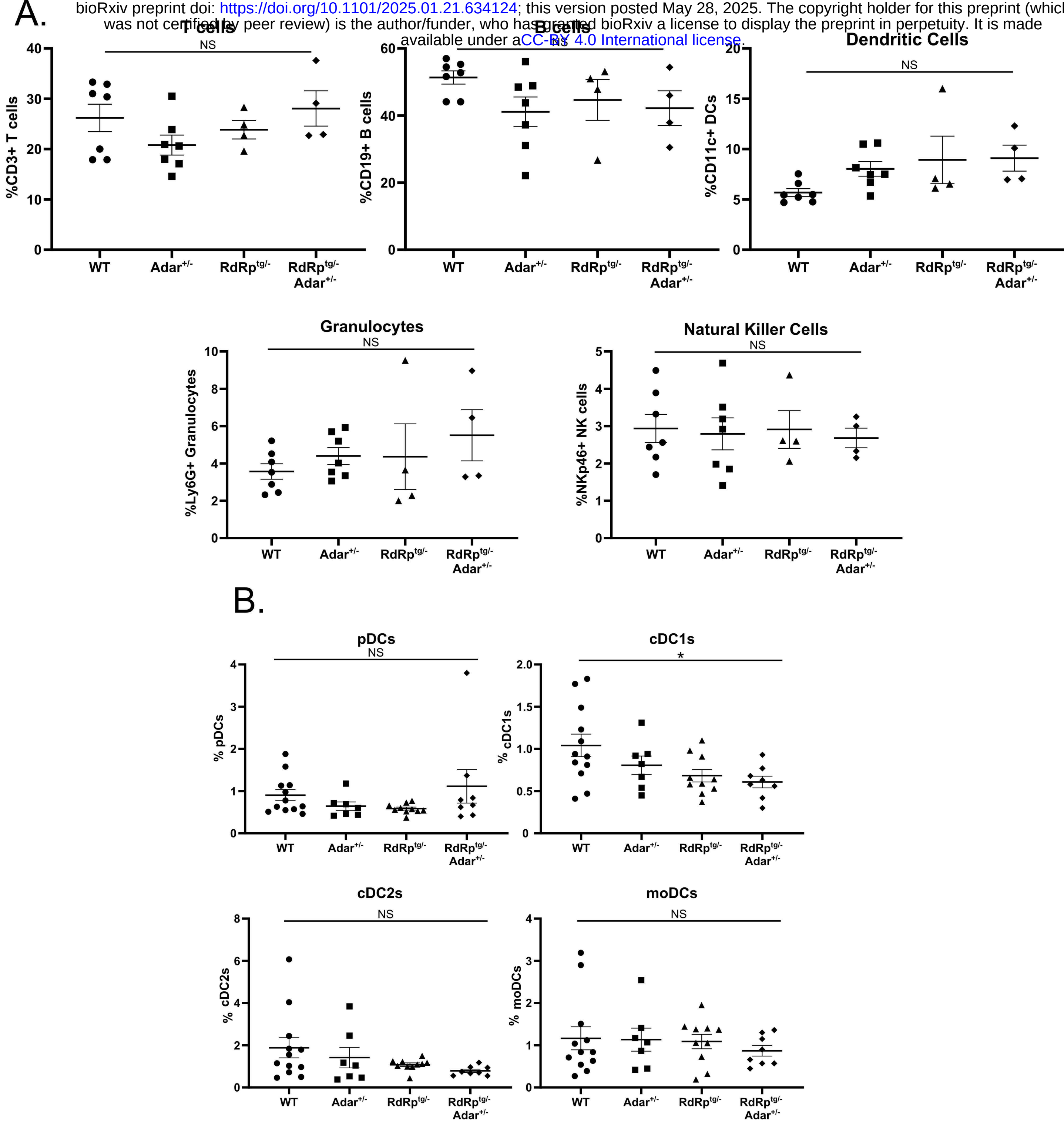

Supplemental Figure 7

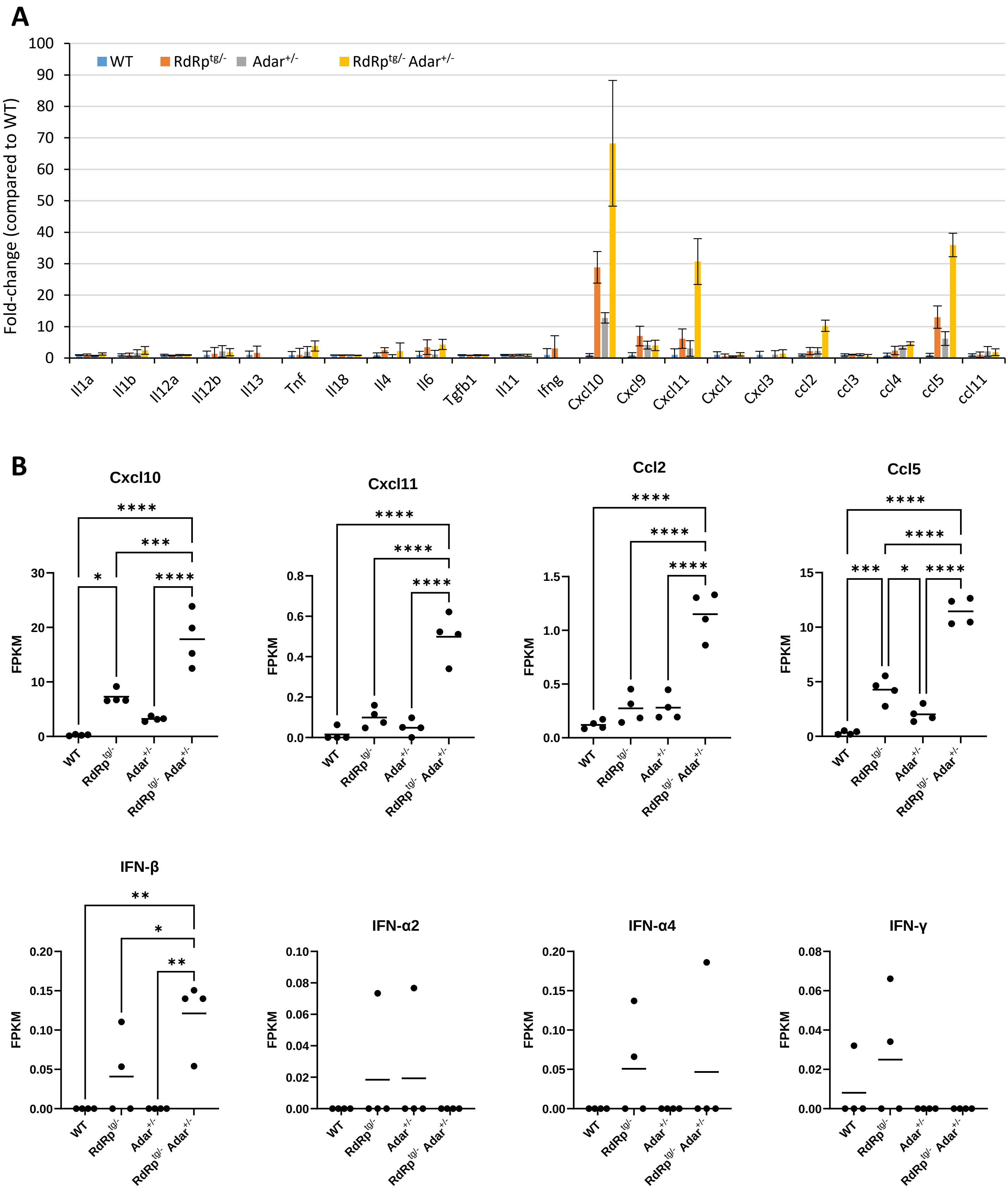

Supplemental Figure 8

bioRxiv preprint doi: https://doi.org/10.1101/2025.01.21.634124; this version posted May 28, 2025. The copyright holder for this preprint (which was not certified by peer review) is the author/funder, who has granted bioRxiv a license to display the preprint in perpetuity. It is made available under aCC-BY 4.0 International license.

	WT vs. RdRp$^{tg/-}$		WT vs. Adar$^{+/-}$		WT vs. RdRp$^{tg/-}$ Adar$^{+/-}$		RdRp$^{tg/-}$ vs. Adar$^{+/-}$		RdRp$^{tg/-}$ vs. Adar$^{+/-}$ RdRp$^{tg/-}$		Adar$^{+/-}$ vs. Adar$^{+/-}$ RdRp$^{tg/-}$	
**Diseases or Functions Annotation**	**-log(B-H p-value)**	**z-score**	**-log(B-H p-value)**	**z-score**	**-log(B-H p-value)**	**z-score**	**-log(B-H p-value)**	**z-score**	**-log(B-H p-value)**	**z-score**	**-log(B-H p-value)**	**z-score**
Antimicrobial response	0.00000	**2.39**	1.97E-09	1.455	NaN	NaN	0.00000	**-2.39**	1.08E-05	NaN	1.21E-12	0.995
Antiviral response	0.00000	**2.39**	2.77E-11	1.455	NaN	NaN	0.00000	**-2.39**	2.82E-06	NaN	6.75E-12	0.995
Apoptosis of breast cancer cell lines	NaN	NaN	NaN	NaN	NaN	NaN	NaN	NaN	NaN	NaN	0.00146	**2.808**
Apoptosis of epithelial cells	NaN	NaN	NaN	NaN	NaN	NaN	NaN	NaN	NaN	NaN	0.00003	**2.55**
Apoptosis of liver cells	NaN	NaN	NaN	NaN	NaN	NaN	NaN	NaN	NaN	NaN	0.00146	**2.169**
Cell death of epithelial cells	0.00251	**2.414**	NaN	NaN	NaN	NaN	NaN	NaN	1.06E-03	0.922	1.41E-05	**3.058**
Demyelination of nerves	NaN	NaN	NaN	NaN	NaN	NaN	NaN	NaN	0.00001	**2.195**	NaN	NaN
Differentiation of Th17 cells	NaN	NaN	NaN	NaN	NaN	NaN			NaN	NaN	0.00014	**-2.382**
Encephalitis	3.09E-04	-1.925	3.72E-07	0.264	NaN	NaN	NaN	NaN	1.09E-04	-1.361	0.00000	**-3.488**
Experimental autoimmune encephalomyelitis	5.09E-04	-1.639	7.57E-07	0.659	NaN	NaN	1.45E-06	1.881	1.66E-04	-1.37	0.00000	**-3.326**
Inflammation of absolute anatomical region	8.46E-06	-1.905	5.51E-08	-0.527	NaN	NaN	1.39E-07	1.365	0.00002	**-2.537**	0.00000	**-3.351**
Inflammation of organ	3.08E-05	-1.264	1.75E-07	0.255	NaN	NaN	1.71E-07	1.078	0.00001	**-2.346**	0.00000	**-2.881**
Inhibition of cells	NaN	NaN	NaN	NaN	NaN	NaN	NaN	NaN	NaN	NaN	0.00142	**2.166**
Maturation of phagocytes	NaN	NaN	2.29E-03	-0.169	NaN	NaN	0.00074	**-2.183**	NaN	NaN	5.47E-04	0.891
Necrosis	8.48E-03	1.117	NaN	NaN	NaN	NaN	NaN	NaN	0.00160	**2.156**	0.00004	**2.291**
Necrosis of epithelial tissue	NaN	NaN	NaN	NaN	NaN	NaN	NaN	NaN	1.36E-04	1.014	0.00000	**2.072**
Neuronal cell death	NaN	NaN	NaN	NaN	NaN	NaN	NaN	NaN	0.00476	**2.302**	0.00006	**2.309**
Proliferation of antigen presenting cells	NaN	NaN	NaN	NaN	NaN	NaN	2.26E-03	-0.865	3.64E-03	NaN	0.00014	**2.201**
Proliferation of blood cells	0.00421	**2.084**	NaN	NaN	NaN	NaN	NaN	NaN	9.98E-04	0.317	8.85E-06	0.535
Proliferation of immune cells	0.00429	**2.084**	NaN	NaN	NaN	NaN	NaN	NaN	9.14E-04	0.358	1.70E-05	0.808
Quantity of blood cells	0.00064	**2.383**	NaN	NaN	NaN	NaN	0.00193	**-2.021**	1.63E-04	0.487	2.79E-06	0.67
Quantity of IL-12 in blood	0.00002	**-2**	6.62E-03	NaN	NaN	NaN	0.00002	**2**	NaN	NaN	0.00007	**-2**
Quantity of interleukin	NaN	NaN	NaN	NaN	NaN	NaN	NaN	NaN	NaN	NaN	0.00064	**-2**
Quantity of leukocytes	0.00046	**2.295**	NaN	NaN	NaN	NaN	1.60E-03	-1.906	1.02E-04	0.167	9.96E-07	0.336
Quantity of phagocytes	0.00104	**2.191**	NaN	NaN	NaN	NaN	2.21E-03	-1.544	2.25E-05	-0.125	5.80E-05	-0.102
Quantity of protein in blood	0.00769	**-2.909**	NaN	NaN	NaN	NaN	0.00034	**2.088**	NaN	NaN	6.48E-04	-1.717
Rheumatic Disease	0.00000	**-2.219**	7.94E-05	-1	NaN	NaN	7.74E-07	1.179	3.07E-05	-0.503	2.99E-08	-1.157

Supplemental Figure 9

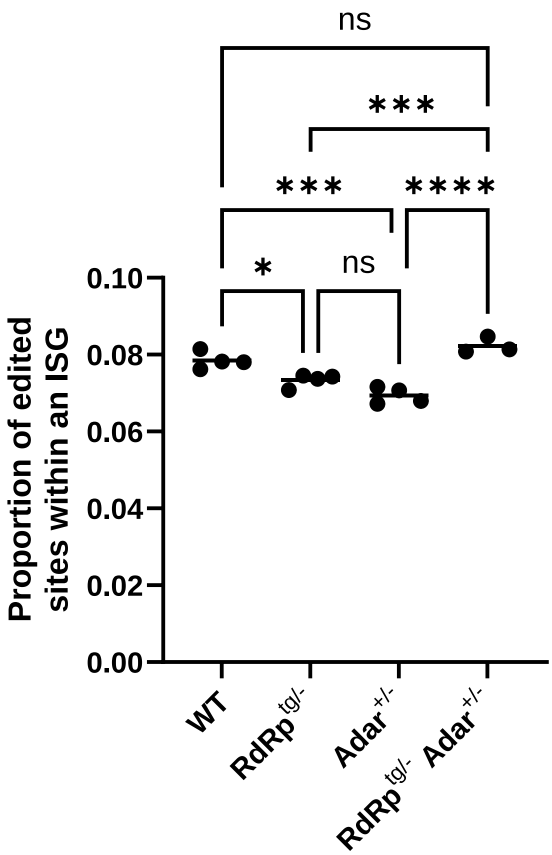

Supplemental Figure 10

bioRxiv preprint doi: https://doi.org/10.1101/2025.01.21.634124; this version posted May 28, 2025. The copyright holder for this preprint (which was not certified by peer review) is the author/funder, who has granted bioRxiv a license to display the preprint in perpetuity. It is made available under aCC-BY 4.0 International license.

Supplemental Figure Legends

Fig. S1. Overall B cell and T cell populations are marginally changed during BM12 transfer. Single cell, RBC-lysed splenocyte suspensions were made from sham or BM12 transferred animals for flow cytometry analysis. **(A)** Overall B cell populations in animals as defined by cd19 expression. **(B)** Overall $CD4^+$ T cell population in the spleens of experimental animals, as defined by TCRβ and CD4 expression. Data were analyzed using a one-way ANOVA followed by a Tukey tests where * = $p < 0.05$, ** = $p < 0.01$, *** = $p < 0.001$, **** = $p < 0.0001$. n = 7, 6, 8, 8 for WT (PBS), *RdRp*$^{tg/-}$ (PBS), WT (BM12), and *RdRp*$^{tg/-}$ (BM12), respectively. Data represent individual animals with the mean and s.d. shown as bars.

Fig. S2. Analysis of organ calcification. Flash frozen brain and formalin-fixed paraffin embedded heart were collected from 6-week-old mice. **(A)** Heart samples were stained with Alizarin Red and calcium depositions were manually counted and shown as Alizarin Red foci per animal (n = 3, 5, 5, 10 for WT, *Adar*$^{+/-}$, *RdRp*$^{tg/}$, *RdRp*$^{tg/-}$ *Adar*$^{+/-}$, respectively). Representative heart section images are shown to the right: (i) positive control heart provided by UCDSOM Research Histology Core Laboratory; (ii) RdRp$^{tg/-}$ Adar$^{+/-}$ heart without Alizarin red foci; (iii) RdRp$^{tg/-}$ Adar$^{+/-}$ heart with a positive focus. **(B)** Calcium was quantified from brain tissues (n = 3, 4, 5, 5). Data were analyzed using a one-way ANOVA followed by a Tukey tests; there were no significant differences between the groups. ns: not significant.

Fig. S3. Heatmap of differentially expressed ISGs across the four genotypes. Enlarged image of Fig. 5B with gene names displayed. FPKM values were log transformed with 1 pseudocount to facilitate visualization.

Fig. S4. Predicted upstream regulators. Predicted upstream signaling regulators as determined by IPA in various genotype comparisons between WT, *Adar*$^{+/-}$, *RdRp*$^{tg/-}$*,* and *Adar*$^{+/-}$ *RdRp*$^{tg/-}$ mice where red/green indicates whether the gene itself is upregulated/downregulated in the RNA-seq and orange/blue indicates predicated activation/inhibition of the molecular pathway it regulates.

Fig. S5. Loss of MDA5 rescues runting phenotype and abolished ISG upregulation. 6-week-old animals were weighed **(A)** and RNA was isolated from whole-brains for qPCR analysis of the ISGs Ifit1 **(B)**, Isg15 **(C)**, and Oasl2 **(D)**. Data were analyzed using a one-way ANOVA followed by a Tukey tests where * = $p < 0.05$, ** = $p < 0.01$, *** = $p < 0.001$, **** = $p < 0.0001$. For (A), n = 4, 5, 6, 10, and 4 for WT, *Adar*$^{+/-}$, *RdRp*$^{tg/}$, *RdRp*$^{tg/-}$ *Adar*$^{+/-}$, and *RdRp*$^{tg/-}$ *Adar*$^{+/-}$ *Ifih1*$^{-/-}$, respectively. For (B-D), n = 4, 3, 5, 5, and 4. Data points represent individual animals with the mean for each group shown as a bar.

Fig. S6. Disease and Biological Functions. Enriched diseases and biological functions across comparisons of gene expression changed between WT, *Adar*$^{+/-}$, *RdRp*$^{tg/-}$*,* and *Adar*$^{+/-}$ *RdRp*$^{tg/-}$ mice. Orange indicated that the disease or biological function is predicted to be enriched for and blue indicated is predicted to be inhibited.

Fig. S7. Certain immune cell populations are unchanged in *RdRp*$^{tg/-}$ *Adar*$^{+/-}$ mice. Splenocytes from 4-5 week old mice were harvested and single cell, RBC-lysed solutions were made for FACS analysis. **(A)** T cells, B cells, DCs, granulocytes, and NK cells show no difference across groups measured. n = 7, 7, 4, and 4 for WT, *Adar*$^{+/-}$, *RdRp*$^{tg/}$, and *RdRp*$^{tg/-}$ *Adar*$^{+/-}$, respectively. **(B)** DC subsets including pDCs, cDC1s, cDC2s, and moDCs show minor or no difference in populations across groups. n = 12, 7, 10, and 8 for WT, *Adar*$^{+/-}$, *RdRp*$^{tg/}$, and *RdRp*$^{tg/-}$ *Adar*$^{+/-}$, respectively. ll data was analyzed using a one-way ANOVA followed by a Tukey

bioRxiv preprint doi: https://doi.org/10.1101/2025.01.21.634124; this version posted May 28, 2025. The copyright holder for this preprint (which was not certified by peer review) is the author/funder, who has granted bioRxiv a license to display the preprint in perpetuity. It is made available under aCC-BY 4.0 International license.

test to determine significance. * = $p < 0.05$. Data represent individual animals, graphs show means and s.d.

Fig. S8. Chemokine, cytokine and IFN expression changes. (A) RNA-seq-derived gene expression changes were determined for chemokines and cytokines and are shown as the average fold-change compared to wild-type mice, +/- s.d. **(B)** Individual animal FPKM values for cxcl10, cxcl11, ccl2, ccl5, IFN-β, IFN-α2, IFN-α4, and IFN-γ were graphed separately. Data were analyzed using a one-way ANOVA followed by a Tukey test to determine significance. No significant changes were observed for IFN-α2, IFN-α4, and IFN-γ. * = $p < 0.05$, ** = $p < 0.01$, *** = $p < 0.001$, **** = $p < 0.0001$.

Fig. S9. Diseases and Biological Functions enriched for in A-to-I edited genes. Genes with A-to-I edit sites and RNA-seq-derived expression changes were analyzed using IPA to determine enriched diseases and biological functions across genotype comparisons (WT, *Adar*$^{+/-}$, *RdRp*$^{tg/-}$, and *Adar*$^{+/-}$ *RdRp*$^{tg/-}$). Orange indicates that the disease or biological function is predicted to be enriched for or activated and blue indicates is predicted to be inhibited.

Fig. S10. Proportion of edited sites in ISG RNAs as a fraction of all uniquely edited sites per animal. Sites of A-to-I editing that were within an ISG were identified and the proportion of ISG edited sites out of all unique edited sites per animal was calculated. Bars indicate group means, dots represent individual animals, and the error bars are s.d. Data were analyzed using a one-way ANOVA followed by a Tukey tests where * = $p < 0.05$, *** = $p < 0.001$, **** = $p < 0.0001$, ns = not significant.

bioRxiv preprint doi: https://doi.org/10.1101/2025.01.21.634124; this version posted May 28, 2025. The copyright holder for this preprint (which was not certified by peer review) is the author/funder, who has granted bioRxiv a license to display the preprint in perpetuity. It is made available under aCC-BY 4.0 International license.

Supplemental Methods, Miller et al.

PCR primers and antibodies

a. Primers

Mus musculus

Gapdh: 5'-CATGGCCTTCCGTGTTCCTA-3' & 5'-CTATGTTTCCCGCGGCACGTCAGATCCA-3'
Ifit1: 5'-TTTGTTGTTGTTGTTGTTCGTT-3' & 5'-GCAGGAATCAGTTGTGATCT-3'
ISG15: 5'- TGGTACAGAACTGCAGCGAG -3' & 5'- CAGCCAGAACTGGTCTTCGT -3'
Oasl2: 5'-CAAACAAACAAACAAACCCTCTC-3' & 5'-TCAAGGTGTCACTCTGCAT-3'
Adar p150 5'-GGCCTTGCCGGCACTATGTCTC-3' & 5' GCGGGTATCTCCACTTGCTATGCTC-3'
Adar p110: 5'-GGTGGAAGACTACGCGTTGGGAC-3' & 5' ACGACTGTGTCTGGTGAGGGAACAC-3'
IFNβ: 5'-CACAGCCCTCTCCATCAACTATAAGCAG-3'& 5'-CTGTAGGTGAGGTTGATCTTTCCATTCAG-3'

Homo sapiens

GAPDH: 5'-TGGACCTGACCTGCCGT-3' & 5'-TGGAGGAGTGGGTGTCGC-3'
ISG15: 5'-GTGGACAAATGCGACGAACC-3' & 5'-ATTTCCGGCCCTTGATCCTG-3'
OASL: 5'-GGATCTTCTCCCACACTCACATCT-3' & 5'-CACCATCAGGATTCTTCACGAA-3'

b. Antibodies

Rabbit polyclonal anti-ADAR1 (1:1,000 dilution; Cell Signaling, 14175)
Mouse anti-tubulin (1:5,000 dilution; Sigma, T5168)
Rabbit anti-Rig-I: mouse tissue blots (1:1,000 dilution, Cell Signaling, 3743)
Mouse anti-ADAR1 (1:500 dilution, Santa Cruz Bio, sc-73408)
Rabbit anti-actin (1:5,000 dilution, Cell Signaling, 4967)
Goat anti-rabbit secondary antibody, horseradish peroxidase -conjugated (1:5,000 dilution; Calbiochem, 401393)
Goat anti-mouse secondary antibody horseradish peroxidase-conjugated (1:5,000 dilution; Invitrogen, 31430)
Rabbit anti-ZBP1/DAI (1:1,000 dilution; Novus Biologicals NBP1-76854)
Rabbit anti-ISG15 (1:1000, Cell Signaling Technologies Cat # 2743)

Quantitative PCR. For qPCR analysis of mouse samples, RNA was extracted from mouse tissue using Trizol reagent (Ambion) according to manufacturer instructions. Reverse transcription PCR (RT-PCR) for cDNA synthesis was conducted using Maxima H Minus First Strand cDNA Synthesis Kit (Thermo Scientific) following manufacturer instructions. QPCR analysis for quantification of cellular transcripts was done using 2X qPCR Green Master Mix (APEX). For qPCR analysis from human cell lines, RNA extractions were done using an RNAeasy kit (Qiagen) according to kit protocol. RT- and qPCR were performed as described above. Transcripts were quantified using the $\Delta\Delta C_T$ method relative to *Gapdh* or β-Actin (*Actb*) transcripts.

Immunoblotting. Cells were homogenized in RIPA buffer and protein content was determined by Bradford reaction (Bio-Rad). 30 µg total protein was loaded per well prepared in 6X reducing SDS sample buffer (Bio-Rad, boil for 5-10 minutes prior to loading). Samples were run on 10% agarose gels (Bio-Rad) and transferred to polyvinylidene difluoride membrane (Bio-Rad). Samples were blocked in 5% milk in tris-buffered saline containing 0.1% Tween20 (TBS-T). Immobilon Crescendo Western HRP substrate (Millipore) was used for visualization of bands and imaged on a ChemiDoc XRS+ Imaging System (Bio-Rad).

Cell culture and siRNA delivery. Triplicate biological replicates were used with three technical replicates each. A549 cell lines (ATTC, CCL-185) were maintained in Dulbecco's Modified

bioRxiv preprint doi: https://doi.org/10.1101/2025.01.21.634124; this version posted May 28, 2025. The copyright holder for this preprint (which was not certified by peer review) is the author/funder, who has granted bioRxiv a license to display the preprint in perpetuity. It is made available under aCC-BY 4.0 International license.

Eagle's Medium (Corning) containing 10% fetal bovine serum and penicillin/streptomycin/L-glutamine (Corning, 100x solution). Doxycycline-inducible RdRp-expressing cells were described previously (1). Two different siRNAs were used in combination for *ADAR* knockdown and were purchased from ThermoFisher Scientific (Catalog # 4390824, IDs s1008, s1009, previously validated). Negative control (ThermoFisher) or anti-*ADAR* siRNA were transfected into cells (50 pmol per well in a 6-well plate, cells seeded at >50% confluence) using lipofectamine RNAimax reagent (ThermoFisher) according to manufacturer instructions. Cells were washed 6 hours post transfection and dox (2 μg/mL) was added at this time. At 48 hours post dox addition, cells were lysed for protein and RNA analysis.

Bioinformatics and Computational Methods. We used a combination of RNA sequencing and exome sequencing to evaluate transcriptome-wide patterns of RNA gene expression and ADAR-mediated A-to-I RNA editing. Specifically, we sequenced four biological replicates from each of the four genotypes for RNA-seq (WT-*Adar*$^{+/+}$, WT-*Adar*$^{+/-}$, *RdRp*$^{tg/-}$ *Adar*$^{+/+}$, and *RdRp*$^{tg/-}$*Adar*$^{+/-}$) and a single sample from each genotype for exome sequencing. Exome sequencing was used to establish a baseline level of germline genetic variation within our mouse colony and was used as background for identifying RNA-edited sites. Library preparation and sequencing for single-end RNA-sequencing and paired-end exome sequencing were performed by BGI (Beijing Genomics Institute). The quality of raw reads was assessed using FASTQC v.0.11.5. We used Trimmomatic v.0.27 to filter out low quality reads that had a mean Phred quality score less than 20. Read quality was then reassessed with FASTQC. We used Hisat2 v.2.1.0 to map reads to the mm10 genome using default single-end parameters for RNA-seq and included the --no-mixed --no-discordant flags for the paired-end exome sequencing. Next we used Samtools v.1.3.1 to convert the sam output files into bam format. Then we used PicardTools v.1.119 to add read groups, remove PCR duplicates, and coordinate sort the bam files. To evaluate RNA-seq gene expression patterns across the four genotypes, we first used Stringtie v.1.3.6 to quantify transcripts abundance, and Stringmerge to merge the output Stringtie GTF files. CuffDiff2 was to identify significantly differentially expressed genes (DEGs) with an FDR of 0.05 and Benjamini-Hochberg multiple test correction. In order to reduce false positives, we also required fragments per kilobase per million reads mapped (FPKM) to be at least four, and at 2-fold differences in expression because of the massive gene expression differences observed in(1, 2). We used CummeRbund and Plotly to visualize our results. The Interferome Database was used to identify which DEGs were known ISGs. Finally, we used the Canonical Pathways and Diseases and Biological Functions analyses in IPA to determine which molecular pathways and biological functions were enriched among our conditions and to predict whether they were likely to be activated or inhibited based on gene expression patterns in our dataset. To identify RNA-edited sites, we used REDItools to call SNPs that were present in the RNA-seq, but not present in the exome sequencing. Because the A-to-I editing performed by ADAR is incorporated as A-to-G changes by sequencers, we looked for differences in A-to-G changes across conditions. We required a minimum of 10x coverage and that each putative RNA-editing SNP be sequenced at least twice to reduce the risk of including sequencing errors. First, we used Samtools mpileup to call SNPs in the exome sequencing data. We used REDItoolsDeNovo to call SNPs A-to-I edited sites in the RNA-seq requiring at least 10x coverage and that each SNP was sequenced at least twice. Putative germline SNPs identified in the exome sequencing were subtracted from the RNA SNPs. The resulting SNP tables were converted into bed format and sorted by coordinates using BedTools and we filtered SNPs to those present in all four biological replicates. We then annotated the SNPs using Homer annotatePeaks.pl to determine the genomic features in which SNPs occur. A-to-I editing patterns were compared across the four genotypes in two ways and visualized using SUMO, 1) whether editing occurred in the same vs. different genomic features, allowing editing sites to occur at different locations within that feature, and 2) whether editing occurred at identical sites. Finally, we used IPA to identify significantly enriched molecular pathways and biological functions associated with edited genes

bioRxiv preprint doi: https://doi.org/10.1101/2025.01.21.634124; this version posted May 28, 2025. The copyright holder for this preprint (which was not certified by peer review) is the author/funder, who has granted bioRxiv a license to display the preprint in perpetuity. It is made available under aCC-BY 4.0 International license.

among the 4 genotypes. The RNA-seq and whole exome sequencing datasets have been deposited to the NCBI sequence read archive (SRA SUB14698103).

Flow Cytometry. Mice were euthanized by CO_2 asphyxiation and subsequent cardiac puncture. Spleens were excised and placed in 15 mL conical tubes containing 3 mL of DMEM containing FBS and antibiotics. Spleen was manually dissociated with a pipette tip and pipetting, then passed through a 40 µM nylon cell strainer. 100 µL from each sample were combined and used for the isotype and unstained controls. Cells were gated to exclude debris, doublets, and dead cells based on viability dye staining. Cell population abundance: samples had at least 1x10^6 cells sorted in the live population and cell types are shown as percent of live cells for all flow cytometry data. Each cell type described had a minimum of about 0.5% meaning all cell types described were observed at a frequency of about 5,000 or more per sample. Analysis of cellular populations was conducted as previously described (3). For staining of splenocyte populations, cells were red blood cell depleted as described (3). For analysis including FoxP3 staining, intracellular transcription factor staining was done using Foxp3/Transcription Factor Staining Buffer Set (eBioscience) according to manufacturer instructions for use in a 96-well plate. DC-specific staining panel has been previously described (3). T_{reg} staining panel consists of the following antibodies/cellular stains: fixable viability dye eFlour780 (eBioscience, 65-0865-14), PE anti-mouse FoxP3 (BioLegend, 126404), PerCP-Cy5.5 anti-mouse CD4 (BioLegend, 100434), Brilliant Violet 421 anti-mouse CD25 (BioLegend, 101923), APC anti-mouse CTLA-4 (BioLegend, 106310), Brilliant Violet 711 anti-mouse GITR (BD Bioscience, 563390), Brilliant Violet 605 anti-mouse ICOS (BioLegend, 313538), Brilliant Violet 480 anti-mouse CD44 (BD Bioscience, 566116) and FITC anti-mouse CD62L (BioLegend, 104406). For analysis of B cells (germinal center and plasmablast) the following antibodies and cellular stains were used: fixable viability dye eFlour780 (eBioscience, 65-0865-14), Brilliant Violet 421 anti-mouse CD138 (BioLegend, 142507), PE anti-mouse Fas (BD Bioscience, 554258), Alexa Fluor 488 anti-mouse GL-7 (BioLegend, 144612). For T_{FH} cell analysis and CD45.1 engraftment, the following antibodies and cellular stains were used: fixable viability dye eFlour780 (eBioscience, 65-0865-14), PerCP-Cy5.5 anti-mouse CD4 (BioLegend, 100434), Brilliant Violett 711 anti-mouse TCRβ (BioLegend, 109243), PE anti-mouse PD-1 (BioLegend, 135205), APC anti-mouse CXCR5 (BioLegend, 145506), and Brilliant Violet 421 anti-mouse CD45.1 (BioLegend, 110732). BST-2 was detected with Pacific Blue anti-mouse BST-2 (BioLegend, 127108). Gating for B cell and T_{FH} cell analysis was described in ref (4). Isotype staining for each panel was done in parallel. Gating was set based on fluorescence minus one controls. Cells were gated to exclude debris, doublets, and dead cells based on viability dye staining. Gating for DC-subtype determination was described in ref. (3). All data was acquired on an LSRII (BD Biosciences) and analysis was done using FlowJo software.

ELISAs. Mouse serum was used for ANA detection via ELISA. For detection of anti-dsDNA antibodies, calf thymus DNA (Sigma, D4522) was digested with S1 nuclease (ThermoFisher, EN0321) to remove any single stranded DNA (ssDNA). DNA was ethanol precipitated and resuspended in nuclease free water. Freeze-thaw cycles were avoided to prevent creation of new ssDNA. Nunc MaxiSorp ELISA plates (Sigma) were coated with Poly-L Lysine (0.01% final, Millipore Sigma) prior to use. Plates were coated overnight with dsDNA (3 µg/mL) in PBS at room temperature and blocked with BSA in PBS (3% final, Sigma). For a standard, anti-dsDNA (Abcam, ab27156) was used between the range of 1 µg/mL – 1.37 ng/mL (3-fold dilutions) and serum samples were initially diluted 1:10 and then as needed. For anti-smAg ELISAs, plates were coated with Sm Ag (3 µg/mL, Genway Biotech, GWB-CA4A65) in PBS overnight. Blocking was done in 1% BSA/PBS. For standard, anti-SmAg (Novus Biologicals, NB600-546) was used between the range of 1 µg/mL – 15.63 ng/mL (2-fold dilutions) and serum samples were diluted 1:10 before use and then as needed. For both ELISAs, standard and serum samples were diluted in sample buffer (0.5% Tween20, 1% BSA in PBS) and plates were washed 3-5 times between incubations with wash solution (0.5% Tween20 in PBS). Plates were incubated with samples/standard for 2 hours at 37°C. Detection antibody used was goat anti-mouse IgG

bioRxiv preprint doi: https://doi.org/10.1101/2025.01.21.634124; this version posted May 28, 2025. The copyright holder for this preprint (which was not certified by peer review) is the author/funder, who has granted bioRxiv a license to display the preprint in perpetuity. It is made available under aCC-BY 4.0 International license.

peroxidase (1:10,000 dilution; Sigma, A2554) and peroxidase was developed used TMB ELISA HRP Substrate kit (Seracare) and Sulfuric acid (4N, Fisher Scientific) according to manufacturer instructions.

Calcium Quantification. Whole-brain tissue was flash frozen at necropsy, homogenized by sonication on ice in 4x volumes of calcium assay buffer (Calcium Assay Kit, Abcam ab102505), and free Ca^{2+} ions were quantified following the manufacturer's methods.

Histopathology. Briefly, approximately 2 cm thick tissue sections of 5 week old mice were fixed in 10% formalin for 24 hours then washed twice and suspended in 70% ethanol. This age was chosen as by this point the mice already showed notable changes in growth but had not yet begun to succumb to disease related mortality. Tissues were paraffin embedded, cut and stained for Alizarin Red or H&E by our Research Histology Core Laboratory. H&E-stained slides were analyzed and scored by the IDEXX BioAnalytics histopathology group and are shown in the figure below. Grading was: 0 no change from WT, 1 minimal, 2 mild, 3 moderate, 4 marked changes. All scores were zero except for one $RdRp^{tg}$ kidney which was scored 1.

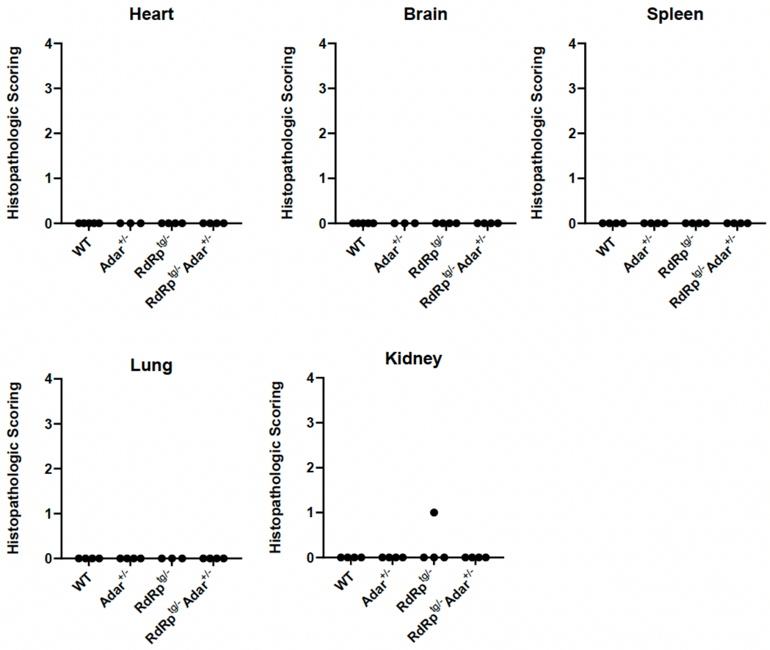

Figure. Histopathological grading of mouse tissues. No significant changes or differences were noted in the $RdRp^{tg/-}$ $Adar^{+/-}$ animals to explain the clinical presentations of this genotype.

References for Supplemental Methods

1. Painter MM, Morrison JH, Zoecklein LJ, Rinkoski TA, Watzlawik JO, Papke LM, et al. Antiviral Protection via RdRP-Mediated Stable Activation of Innate Immunity. *PLoS pathogens.* 2015;11(12):e1005311.
2. Bankers L, Miller C, Liu G, Thongkittidilok C, Morrison J, and Poeschla EM. Development of interferon-stimulated gene expression from embryogenesis through adulthood, with and without constitutive MDA5 pathway activation. *Journal of Immunology.* 2020;204(10):2791-807.
3. Miller CM, Barrett BS, Chen J, Morrison JH, Radomile C, Santiago ML, et al. Systemic Expression of a Viral RdRP Protects against Retrovirus Infection and Disease. *Journal of virology.* 2020;94(9).
4. Klarquist J, and Janssen EM. The bm12 Inducible Model of Systemic Lupus Erythematosus (SLE) in C57BL/6 Mice. *J Vis Exp.* 2015(105):e53319.
